# 

**Published:** 1916-11

**Authors:** 


### CONTENTS.

ORIGINAL COMMUNICATIONS......342
EDITORIAL......359
SELECTIONS AND ABSTRACTS......361
BOOK REVIEW......373

